# DNA Hypomethylation Underlies Epigenetic Swapping between *AGO1* and *AGO1-V2* Isoforms in Tumors

**DOI:** 10.3390/epigenomes8030024

**Published:** 2024-06-22

**Authors:** Jean S. Fain, Camille Wangermez, Axelle Loriot, Claudia Denoue, Charles De Smet

**Affiliations:** 1 Group of Genetics and Epigenetics, de Duve Institute, Université Catholique de Louvain, 1200 Brussels, Belgium; jean.fain@uclouvain.be (J.S.F.); camille.wangermez@uclouvain.be (C.W.);; 2 Group of Computational Biology and Bioinformatics, de Duve Institute, Université Catholique de Louvain, 1200 Brussels, Belgium; axelle.loriot@uclouvain.be

**Keywords:** DNA methylation, cancer epigenetics, cancer-germline gene, Argonaute protein

## Abstract

Human tumors progress in part by accumulating epigenetic alterations, which include gains and losses of DNA methylation in different parts of the cancer cell genome. Recent work has revealed a link between these two opposite alterations by showing that DNA hypomethylation in tumors can induce the expression of transcripts that overlap downstream gene promoters and thereby induce their hypermethylation. Preliminary in silico evidence prompted us to investigate if this mechanism applies to the locus harboring *AGO1*, a gene that plays a central role in miRNA biogenesis and RNA interference. Inspection of public RNA-Seq datasets and RT-qPCR experiments show that an alternative transcript starting 13.4 kb upstream of *AGO1* (*AGO1-V2*) is expressed specifically in testicular germ cells, and becomes aberrantly activated in different types of tumors, particularly in tumors of the esophagus, stomach, and lung. This expression pattern classifies *AGO1-V2* into the group of “Cancer-Germline” (CG) genes. Analysis of transcriptomic and methylomic datasets provided evidence that transcriptional activation of *AGO1-V2* depends on DNA demethylation of its promoter region. Western blot experiments revealed that *AGO1-V2* encodes a shortened isoform of AGO1, corresponding to a truncation of 75 aa in the N-terminal domain, and which we therefore referred to as “∆NAGO1”. Interestingly, significant correlations between hypomethylation/activation of *AGO1-V2* and hypermethylation/repression of *AGO1* were observed upon examination of tumor cell lines and tissue datasets. Overall, our study reveals the existence of a process of interdependent epigenetic alterations in the *AGO1* locus, which promotes swapping between two AGO1 protein-coding mRNA isoforms in tumors.

## 1. Introduction

In many organisms, the transmission of cellular phenotypes is influenced by DNA methylation, a chemical modification of DNA corresponding to the addition of a methyl group on cytosines in cytosine-guanine (CpG) dinucleotides [1]. When present on gene promoters, DNA methylation imposes transcriptional repression. Since methylated CpGs are preserved throughout cell divisions, DNA methylation contributes to perpetuating specific gene expression programs in different cell lineages.

DNA methylation profiles often become altered in the genome of cancer cells [2]. Gains (DNA hypermethylation) are observed in CpG-rich genomic regions that normally remain unmethylated (termed CpG islands), and this can lead to the stable repression of tumor suppressor genes [3]. At the same time, many cancer cells exhibit DNA hypomethylation in other parts of their genome [4]. DNA hypomethylation has been linked to genome instability [5] and the ectopic activation of tissue-specific genes [6]. Many of the genes that become activated following DNA hypomethylation in tumors belong to the group of “cancer-germline” (CG) genes, as their expression is normally restricted to testicular germ cells [7]. CG genes become activated in a significant fraction (10–40%) of tumors of various histological types, and some of them appear to encode proteins with pro-malignant functions [8,9].

Recently, we discovered that the activation of CG genes in tumors can also have epigenetic consequences in cis. We found, indeed, that in some instances, DNA hypomethylation induces the transcription of a long CG gene, which overlaps a downstream gene and causes DNA hypermethylation of its promoter region [10]. This observation revealed an unsuspected connection between DNA hypomethylation and DNA hypermethylation in tumors. DNA hypermethylation of the overlapped downstream promoter appeared to be associated with the well-described process of transcription-mediated deposition of H3K36me3, a histone modification known to attract DNMT3 DNA methyltransferases [11,12,13,14]. Indeed, the body of actively transcribed genes generally shows a high level of DNA methylation, and it was shown that this prevents illegitimate transcription starts within the gene while increasing its level of transcription in some cases [15,16,17,18].

An in silico search identified several genomic loci that potentially undergo dual DNA hypo/hypermethylation alterations in tumor cells. Detailed analysis of two such loci (*GABRA3* and *RERG*) confirmed that the hypomethylated/activated overlapping genes (*CT-GABRA3* and *CT-RERG*, respectively) exhibit features of CG genes. The corresponding transcripts, however, were found to be non-coding, due to the presence of short upstream ORFs (uORFs) in their specific 5′ exons that inhibit translation [10]. The net effect resulting from the activation of these CG genes was therefore exclusively the repression of the overlapped downstream genes. In the *RERG* locus, the CG gene was found to repress two downstream genes (*PTPRO* and *RERG*) known to exert tumor-suppressor functions [19,20].

During the in silico screening we carried out, the genomic region harboring the *AGO1* gene emerged as a potential locus undergoing the process of dual DNA hypo/hypermethylation in tumor cells [10]. AGO1 (Argonaute 1), like the other family members (AGO2-4), plays essential roles in several steps of the RNA interference process, from the biogenesis of mature miRNAs and siRNAs to the execution of interference mechanisms. The mode of interference induced by AGO1 varies according to the cellular location of the target RNA. In the cytoplasm, AGO1 recruits effector proteins that inhibit translation and induce degradation of the targeted mRNAs [21]. In the nucleus, siRNAs direct AGO1 towards specific genomic regions by interacting with nascent RNA transcripts, leading to the local recruitment of chromatin-modifying enzymes. Hence, nuclear AGO1 acts as a regulator of gene transcription and contributes to shaping chromatin architecture [22,23]. In addition, nuclear AGO1 appears to modulate the spliceosome, which may influence cell fate, including in immune cell subtypes [24]. The potential contribution of AGO proteins to tumorigenesis is still unclear, as both tumor-promoting and tumor-suppressor functions have been documented [25].

In the present study, we carried out a detailed analysis of the *AGO1* locus, with the aim to characterize the transcript variant that overlaps the *AGO1* gene, determine its expression and DNA methylation profile in normal and cancerous tissues, study its ability to be translated into a protein, and check whether its activation correlates with epigenetic repression of the downstream *AGO1* gene.

## 2. Results

### 2.1. Specific Expression of the Overlapping mRNA Variant (AGO1-V2) in Testicular Germ Cells

**Figure 1:**
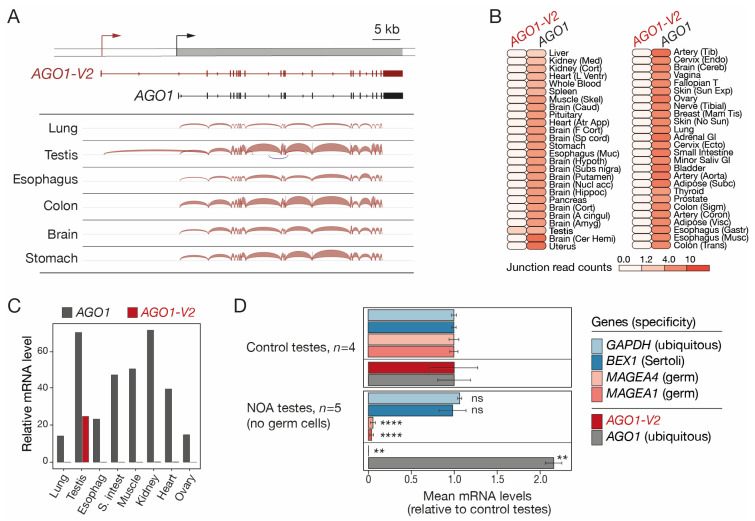
*AGO1-V2* structure and germline-specific expression. (**A**) RNA-Seq data of tissues were analyzed with IGV. Positions of transcription start sites (broken arrows) and exons (boxes) of the two transcript isoforms are depicted. (**B**) Expression profiles of *AGO1-V2* and *AGO1* mRNAs in normal human tissues, based on GTEx junction expression analyses. (**C**) RT-qPCR analysis of *AGO1-V2* and *AGO1* mRNAs (normalized to *ACTB* mRNA levels × 10,000). (**D**) Mean relative mRNA levels of indicated genes were calculated from RNA-Seq data of control and germ cell-free NOA testis samples. Student’s *t*-test with adjusted *p* value (****: *p* ≤ 0.0001; **: *p* ≤ 0.01; ns: not significant).

To better characterize the structure and tissue-specificity of the transcript variant that overlaps AGO1, public RNA-seq data of human testis and five purely somatic tissues (brain, colon, esophagus, lung, and stomach) were downloaded and examined with the Splice Junction analysis tool of the Integrative Genome Viewer (IGV). An alternative transcript starting 13.4 kb upstream of the start site of the reference *AGO1* gene was detected in the testis, but not in the somatic tissues that were analyzed (Figure 1A). This longer transcript contains a specific 5′-exon joined by splicing to all *AGO1* exons except exon 1 and corresponds to an mRNA variant referred to as *AGO1-V2* in the NIH GeneBank database (Figure 1A). To further assess the profile of expression of *AGO1-V2*, RNA-seq data from a large collection of human tissues (GTEx) were examined, focusing on exon junction sequences specific to each of the two *AGO1* transcript isoforms. The results showed that whereas *AGO1* is expressed ubiquitously, *AGO1-V2* displays specific expression in the testis (Figure 1B). To further validate this observation, we designed PCR primers that specifically amplify either *AGO1* or *AGO1-V2* sequences and used them in RT-qPCR experiments on several human tissues. The results confirmed the specific expression of *AGO1-V2* in testis (Figure 1C).

We next sought to determine if the *AGO1-V2* variant is expressed in somatic and/or germ cells in the testis. Single-cell RNA-seq generally provides little information on transcript variant usage. We therefore decided to analyze bulk RNA-seq data of testicular samples isolated from either control subjects or patients with non-obstructive azoospermia (NOA), where germ cell counts are sometimes very low [26]. Exon quantification was performed to compare relative *AGO1* and *AGO1-V2* mRNA levels in control testes (set at 1) versus NOA testes that lack germ cells, as indicated by the reduced expression of *MAGEA1* and *MAGEA4* germ cell markers (Figure 1D). *AGO1-V2* mRNA was found to be strongly reduced in these NOA testes, implying that its expression is restricted to germ cells (Figure 1D). This was not the case for *AGO1*, which, unexpectedly, displayed enhanced expression in NOA testes. Together, our results indicate that, unlike *AGO1*, *AGO1-V2* displays highly specific expression in testicular germ cells.

### 2.2. Transcriptional Activation of AGO1-V2 mRNA in Human Tumors

**Figure 2:**
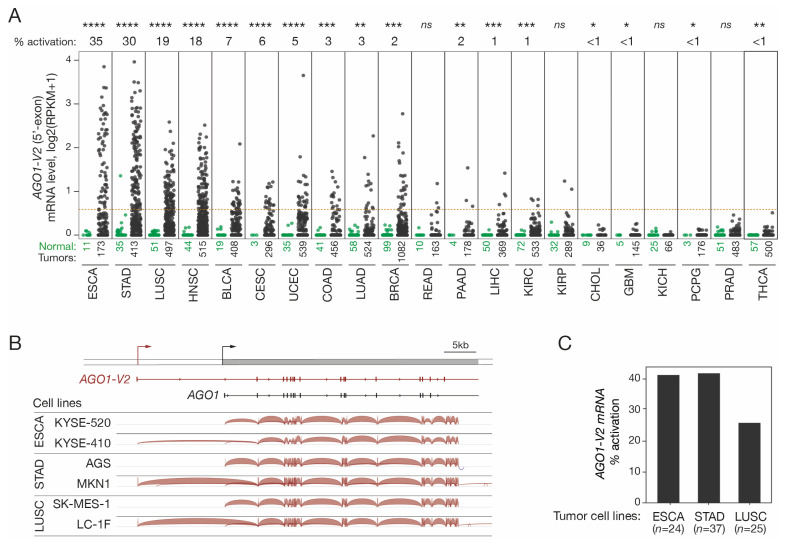
*AGO1-V2* expression analysis in tumor tissues and cell lines. (**A**) RNA-Seq data from the TCGA were downloaded and reads matching the specific 5′-exon of *AGO1-V2* were counted to evaluate its expression level: dots represent log2(RPKM + 1) in each of the normal (green) and tumor (black) tissue samples. Numbers below indicate number of samples. Tumor types are ordered according to frequency (indicated above the graph) of *AGO1-V2* transcriptional activation (considered positive when RPKM was ≥0.5, as indicated by the red dotted line). Welch’s *t*-test with adjusted *p* value was used to analyze differences in expression levels between normal and tumors (****: *p* ≤ 0.0001; ***: *p* ≤ 0.001; **: *p* ≤ 0.01; *: *p* ≤ 0.05; ns: not significant). (**B**) IGV analysis of RNA-Seq data from tumor cell lines that do not or do show expression of *AGO1-V2*. (**C**) Frequency (%) of *AGO1-V2* activation in ESCA, STAD, and LUSC tumor cell lines was inferred from RNA-Seq data of the CCLE, using the same threshold as in (**A**).

The possibility that *AGO1-V2* transcription could become derepressed in tumors was examined. To this end, we first analyzed RNA-seq datasets from various tumor types and corresponding control tissues made accessible by the Cancer Genome Atlas (TCGA). Quantification of the 5′-exon of *AGO1-V2* was performed in order to distinguish this specific mRNA species from other *AGO1* variants (Figure 2A). The results revealed that *AGO1-V2* is indeed aberrantly activated in various types of tumors, with the highest activation frequencies in esophageal squamous cell carcinoma (ESCA, 35%), stomach adenocarcinoma (STAD, 30%), lung squamous cell carcinoma (LUSC, 19%), and head and neck carcinoma (HNSC, 18%).

To ensure that *AGO1-V2* mRNAs produced in tumor cells have the same structure as those described in the testis, RNA-seq data from several tumor cell lines (ESAC, STAD, and LUSC) were downloaded and analyzed with the IGV tool to determine exon usage in *AGO1-V2* positive and negative samples. The results showed that *AGO1-V2* transcripts expressed in tumor cells exhibit the same structure as those in testicular germ cells (Figure 2B). Moreover, activation frequencies in tumor cell lines appeared similar to those observed in vivo in tumor tissues (Figure 2C). Overall, our observations show that *AGO1-V2* has an expression profile, namely in testicular germ cells and certain tumors, which enables it to be classified in the group of cancer-germline (CG) genes.

### 2.3. AGO1-V2 Encodes a Truncated Protein Isoform

**Figure 3:**
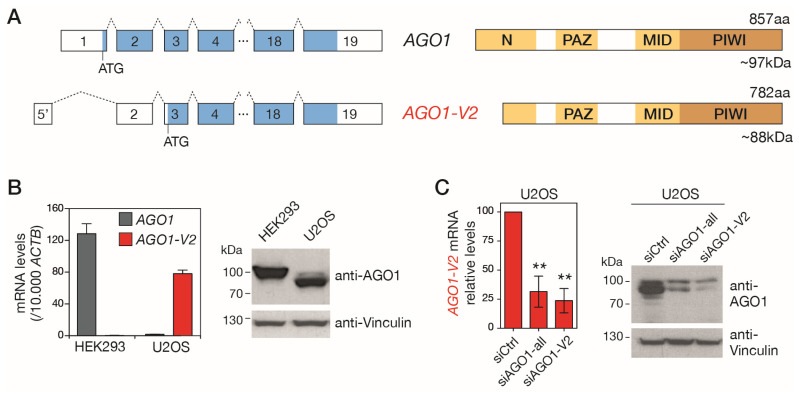
*AGO1-V2* encodes a truncated isoform of AGO1. (**A**) Schematic depiction of *left*, *AGO1* and *AGO1-V2* exons (boxes) and ORFs (bleu); *right*, expected domains (yellow) in the encoded protein. (**B**) Two cell lines expressing either *AGO1* (HEK293) or *AGO1-V2* (U2OS), as shown by RT-qPCR experiments, were used for Western blot analysis using antibodies directed against the C-terminal portion of AGO1 (anti-Vinculin was used as loading control). (**C**) U2OS cells were transfected with control siRNAs or siRNAs directed against all *AGO1* mRNA variants or only *AGO1-V2* (inhibition validated by RT-qPCR, *n* = 3, **: *p* ≤ 0.01, ANOVA test), and submitted to Western blot as in (**B**).

Although *AGO1-V2* is predicted in gene databases to encode a shorter isoform of the AGO1 protein (Figure S1), there is currently no experimental evidence for the existence of this protein. Examination of the *AGO1-V2* mRNA sequence from its 5′ end indeed reveals the presence of an ATG in exon 3 that complies with Kozak’s rules. This start codon is expected to initiate the translation of a truncated isoform of AGO1, lacking the first 75 amino acids (Figure 3A). Missing amino acids are in the N domain, a segment of AGO1 involved in miRNA maturation and target RNA recognition [27,28]. The molecular mass of the truncated protein (AGO1 isoform 2) is expected to be 88 kDa, whereas that of the full-length AGO1 protein (AGO1 isoform 1) is 97 kDa. We took advantage of this size difference to distinguish isoform 2 of AGO1 in Western blot experiments and thus verify its existence. First, tumor cell lines available in the lab were tested using RT-qPCR in order to identify those that express one or the other *AGO1* mRNA variant (Figure S2). This led to the identification of HEK293, which expresses only *AGO1*, and U2OS, where we observed the predominant expression of *AGO1-V2* (Figure 3B).

Proteins extracted from these cell lines were submitted to Western blot analysis with antibodies directed against the C-terminal portion of AGO1, which is comprised of both isoform 1 and isoform 2. As shown in Figure 2B, while a band with a size (~100 kDa) corresponding to AGO1 isoform 1 was present in HEK293, a band of reduced size, compatible with the expected size of AGO1 isoform 2, was visible in U2OS. To confirm that this lower band was indeed the product of *AGO1-V2*, we performed additional Western blots using U2OS cells in which this variant was downregulated by siRNAs targeting either its specific 5′ exon (siAGO1-V2) or exons common to the two AGO1 variants (siAGO1-all). The results showed that the inhibition of *AGO1-V2* mRNA is indeed accompanied by a reduction in the intensity of the anti-AGO1-labelled band of around 88 kDa (Figure 3C). Together, these observations confirm that the *AGO1-V2* transcript is translated into a shortened protein isoform of AGO1, matching the size of the predicted isoform 2, which we propose to name “∆NAGO1”.

### 2.4. DNA Demethylation Accounts for AGO1-V2 Transcriptional Activation

**Figure 4:**
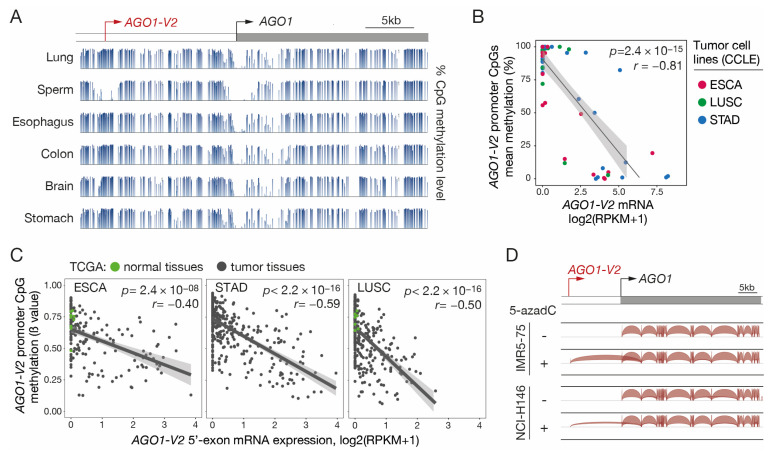
*AGO1-V2* transcription is correlated with promoter DNA demethylation. (**A**) Bisulfite-Seq data (NIH Roadmap epigenomics) revealing the methylation level of CpG sites (histograms) surrounding *AGO1-V2* and *AGO1* TSS (broken arrows) in normal tissues. (**B**) Methylomic (RRBS) and transcriptomic (RNA-Seq) datasets of indicated types of tumor cell lines (CCLE) were downloaded to evaluate correlation between levels of *AGO1-V2* promoter methylation (% methylation) and mRNA expression (log2(RPKM + 1)). Pearson’s correlation coefficient (*r*) with *p*-value (*p*) and linear regression line are shown. (**C**) Tumor tissue samples were analyzed as in B, except that datasets from the TCGA interrogate the methylation level (ß value) of only one CpG in the *AGO1-V2* promoter. Each dot corresponds to a normal (green) or tumor (black) tissue sample. (**D**) RNA-Seq data from two tumor cell lines treated or not with 5-aza-2′-deoxycytidine (5-azadC) were downloaded and analyzed with IGV. Positions of *AGO1-V2* and *AGO1* TSS are indicated (broken arrows).

Since many CG genes use DNA methylation as a primary mechanism of transcriptional regulation, we decided to verify whether this is also the case for *AGO1-V2*. Inspection of the sequence surrounding the *AGO1-V2* transcription start site (TSS) with the GC Profile 2.0 web tool revealed the presence of a CpG-rich island between positions −224 and +72 (Figure S3). To verify the involvement of DNA methylation in regulating *AGO1-V2* expression, we first analyzed bisulfite-seq datasets (NIH Roadmap epigenomics) to determine the CpG methylation profile in the entire *AGO1* locus in various human somatic tissues and in sperm. We observed that CpGs located around the *AGO1-V2* TSS were mostly unmethylated in sperm, and instead, were highly methylated in all somatic samples (Figure 4A). In contrast, the TSS of AGO1 was surrounded by a stretch of CpGs that appeared unmethylated in all tissues.

To further establish a role of DNA methylation in *AGO1-V2* regulation, RNA-seq and reduced representation bisulfite sequencing (RRBS) datasets from ESCA, STAD, and LUSC tumor cell lines (CCLEs) were downloaded and analyzed to compare levels of mRNA expression and mean promoter DNA methylation. The results revealed a strong correlation between *AGO1-V2* expression and promoter demethylation in the analyzed tumor cell lines (Figure 4B). Moreover, examination of omic datasets provided by the TCGA revealed that a similar correlation pertains in vivo in tumor tissue samples originating from ESCA, STAD, and LUSC (Figure 4C).

Finally, experimental evidence of the role of DNA methylation in regulating *AGO1-V2* transcription was provided by the observation that the transcript was induced upon treatment with a DNA methylation inhibitor (5-aza-2′-deoxycytidine) in initially non-expressing tumor cell lines (Figure 4D). Together, these data suggest that DNA methylation acts as a determining mechanism of control of *AGO1-V2* expression, and that transcriptional activation of this variant in tumors depends on demethylation of CpGs within its 5′-region.

### 2.5. Hypomethylation/Activation of AGO1-V2 Correlates with Hypermethylation/Repression of AGO1

**Figure 5:**
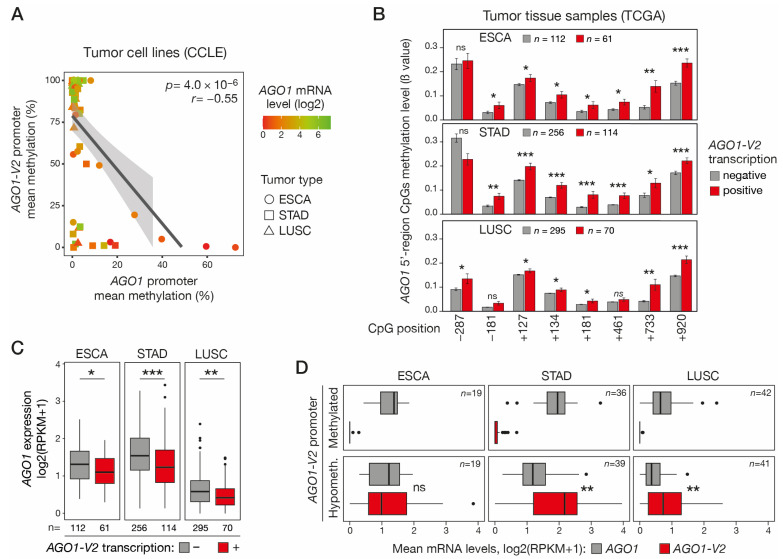
Activation of *AGO1-V2* is correlated with *AGO1* promoter hypermethylation and transcriptional repression. (**A**) Methylomic datasets (RRBS, CCLE) were downloaded to evaluate correlation between *AGO1-V2* and *AGO1* promoter mean methylation levels (%) in each tumor cell line. Pearson’s correlation coefficient (*r*) with *p*-value (*p*) and linear regression line are shown. A color code indicates the level of *AGO1* mRNA expression (log2(RPKM + 1)) in tumor cell lines. (**B**) Tumor tissue samples (TCGA) of indicated types were divided into two groups according to *AGO1-V2* expression status (positive: RPKM ≥ 0.5; negative: <0.5), and mean methylation levels (ß values) of CpGs surrounding *AGO1* start site (position relative to TSS) were compared. One-tailed Welch’s *t*-test with adjusted *p* value was used to assess significance of differences (***: *p* ≤ 0.001; **: *p* ≤ 0.01; *: *p* ≤ 0.05; ns: not significant). (**C**) TCGA tumor tissue samples were divided into *AGO1-V2* positive and negative groups as described in B, and mean *AGO1* mRNA expression levels were compared. One-tailed Student’s *t*-test, adjusted *p* value (***: *p* ≤ 0.001; **: *p* ≤ 0.01; *: *p* ≤ 0.05). (**D**) For each indicated tumor type, tumor tissue samples (TCGA) were divided into two groups according to *AGO1-V2* promoter CpG methylation status (hypomethylated: 10th percentile of ß value; methylated: 90th percentile), and mean mRNA expression levels (log2(RPKM + 1)) of either *AGO1* or *AGO1-V2* were compared. Paired *t*-test, adjusted *p* value (**: *p* ≤ 0.01).

We next sought to determine if DNA hypomethylation and activation of *AGO1-V2* could be associated with DNA hypermethylation and repression of the downstream *AGO1* gene, as this was suggested by previous preliminary data in lung adenocarcinoma cell lines [10]. To this end, we analyzed RRBS data from the CCLE to compare *AGO1-V2* and *AGO1* promoter methylation levels in ESCA, STAD, and LUSC tumor cell lines. The results showed a significant correlation between hypomethylation of the *AGO1-V2* promoter and hypermethylation of the downstream *AGO1* promoter (Figure 5A). Consistent results were obtained in vivo in ESCA, STAD, and LUSC tumor tissues, where transcriptional activation of *AGO1-V2* was found to be associated with increased methylation of all or most CpGs that were probed in the *AGO1* promoter region (Figure 5B). Promoter DNA hypermethylation is classically linked to transcriptional repression. Consistently, *AGO1* mRNA levels were generally low in tumor cell lines with a hypermethylated *AGO1* promoter (Figure 5A). Moreover, *AGO1-V2* transcriptional activation in vivo in ESCA, STAD, and LUSC tissue samples was significantly associated with decreased AGO1 mRNA levels (Figure 5C). Together, these observations indicate that DNA hypomethylation and activation of *AGO1-V2* in tumor cells is often accompanied by DNA hypermethylation and the repression of *AGO1*. The consequence of this coupling process is that in tumors where the *AGO1-V2* gene is epigenetically activated, its mRNA levels equal or exceed that of *AGO1* (Figure 5D), thereby favoring swapping between the two isoforms.

## 3. Discussion

Our study provides a new example of the previously described process of in cis coupled epigenetic alteration, in which the hypomethylation of one DNA segment promotes the hypermethylation of neighboring DNA sequences via the activation of an overlapping transcript [10]. The present findings therefore strengthen the potential importance of this mechanism as a source of epigenetic alterations in the genome of cancer cells.

In the genomic loci we studied previously (*GABRA3* and *RERG*), the overlapping transcripts were non-coding, and therefore their activation in tumors merely inhibited the expression of the downstream hypermethylated genes. Here, we demonstrate that in the *AGO1* locus, DNA hypomethylation causes the activation of a transcript, *AGO1-V2*, which not only overlaps the downstream *AGO1* gene and favors its repression but also produces a different protein isoform (∆NAGO1). The process of coupled hypo/hypermethylation thus promotes swapping between the two protein-coding mRNA isoforms by ensuring that the expression of one (*AGO1-V2*) is accompanied by the downregulation of the other (*AGO1*).

Previous studies showed that modulating the ratio between different isoforms of the “same” protein can have functional consequences and participate in tumor progression [29]. A well-described example is p53, where alterations in the ratio between two isoforms (∆40p53 and p53α) have been shown to influence prognosis and therapeutic response in breast cancer [30,31]. It would therefore be interesting to assess whether the change in the ratio between transcripts encoding AGO1 and ∆NAGO1 that we observed in several tumors could affect cellular functions and malignancy.

The ∆NAGO1 protein isoform we detected lacks 75 aa in the N domain of AGO1. The N domain of Argonaute proteins is known to play a key role during miRNA biogenesis by initiating the unwinding of small RNA duplexes, before the elimination of one of the two RNA strands from the RISC complex [32]. The N domain also appears to influence the efficiency with which the mature miRNA-RISC complex exerts its interference activity on target RNAs [28,33]. It is therefore to be anticipated that a shift in the ratio between AGO1 and ∆NAGO1 could lead to changes in the miRNA repertoire of the cell and modulate its interference network. Of note, a number of studies reported extensive changes in miRNA and siRNA contents in cancer cells, which were associated with tumor development [34,35,36]. It has also been described that germ cells demarcate from somatic cells via the expression of a unique profile of small interfering RNAs [37,38]. In-depth investigations into the specific functions of ∆NAGO1 are awaited and will help to understand how this protein isoform could influence the biology of germ and tumor cells.

Recently, another AGO1 isoform, AGO1x, has been detected in several human tissues, particularly in the brain [39]. AGO1x is a C-terminally elongated isoform of AGO1 resulting from a process of translational readthrough that adds 33 aa beyond the canonical stop codon [39]. This isoform of AGO1 was shown to impede the miRNA effector pathway [39] and moderate the accumulation of dsRNAs in cells, thereby lowering interferon-induced apoptosis [40]. Interestingly, breast cancer cells that escaped therapy-induced senescence showed increased expression of AGO1x, and this was correlated with higher cell proliferation [40,41].

Our analyses indicate that the transcriptional activation of *AGO1-V2* does not always lead to hypermethylation of the downstream *AGO1* promoter. This was observed in several tumor samples and in the testis. It therefore appears that overlapping transcription does not inevitably induce DNA hypermethylation of the downstream promoter, but rather increases the risk that it becomes hypermethylated. The same conclusion was reached when *GABRA3* and *RERG* loci were analyzed [10]. Although the ability of overlapping transcription to induce promoter hypermethylation is well documented [12,13,42,43], it is known that mechanisms exist to protect gene promoters from de novo methylation, especially promoters exerting high transcriptional activity [12,44]. The ubiquitous nature of *AGO1* transcription likely explains the partial resistance of its promoter to hypermethylation. Such resistance can, however, become compromised in tumors, where mechanisms of protection against de novo methylation are altered, often as a consequence of exacerbated activity of DNA methyltransferases or reduced function of DNA demethylases [45,46,47,48].

In conclusion, our work has uncovered a new CG gene and demonstrated that it produces a novel isoform of Argonaute proteins, which are known contributors to RNA interference processes. It is, therefore, reasonable to pursue the study of this gene, for instance, by performing in situ detection experiments and in vivo functional assays, with the hope that it may, in the future, be of diagnostic or therapeutic interest for cancer. Our analyses also reveal a new example where DNA hypomethylation and the activation of a CG gene are accompanied by DNA hypermethylation and repression of a downstream promoter and indicate that, in this case, this dual epigenetic process favors swapping between two protein-encoding mRNA isoforms.

## 4. Material and Methods

### 4.1. Cell Culture

Human osteosarcoma (U2OS) and embryonic kidney (HEK293) cell lines, which were selected on the basis of AGO1-V2 expression status (Figure S2), were kindly provided by A. Decottignies and B. Lauwerys (Université catholique de Louvain), respectively. They were cultured in DMEM (Life Technologies, Thermo Fisher, Rockford, IL, USA) medium, supplemented with 10% of fetal bovine serum (FBS, Sigma, Burlington, MA, USA), 1% non-essential amino acids (Life Technologies), and 1% penicillin/streptomycin (Life Technologies). For passaging and harvesting, U2OS cells were detached with Trypsin-EDTA (0.05%, Gibco, Thermo Fisher) and HEK293 cells with PBS 1X-EDTA (0.5 M).

### 4.2. siRNAs Transfection

The “siAGO1-all” siRNA was obtained from Dharmacon (L004638-00-0020, Horizon Discovery, Lafayette, CO, USA) and consisted of a pool of 4 siRNAs directed against all *AGO1* mRNA variants, whereas the “siAGO1-V2” siRNA was designed to specifically target *AGO1-V2* mRNA (exon-5′) and was synthesized commercially (Kaneka Eurogentec, Seraing, Belgium). For transfection with siRNAs, U2OS cells were seeded at 300,000 cells/well of a 6w plate and were exposed to siAGO1-all or siAGO1-V2 or a control siRNA directed against luciferase (siLuc) at a final concentration of 100 nM, using Lipofectamine 2000 and applying the reverse transfection protocol provided by the manufacturer (Invitrogen, Waltham, MA, USA). Cells were harvested on day 3 post-transfection for RNA and protein analysis. siRNA sequences are listed in Table S1.

### 4.3. RT-qPCR Analyses

RNA of normal tissue samples (lung, testis, esophagus, colon, brain, and stomach) was purchased from Ambion (Thermo Fisher). RNA of normal and tumor cell lines was extracted using TriPure isolation reagent (Roche, Basel, Switzerland). Reverse transcription was performed using the MML-V reverse transcriptase kit (Invitrogen, Thermo Fisher), random hexamers (Invitrogen), the Ribolock RNAse inhibitor (Invitrogen, 20U), and 2 μg of total RNA per reaction in a final volume of 20 μL. qPCR analyses were performed using the KAPA SYBR FAST kit (Sigma-Aldrich Merck, Darmstadt, Germany) with 1/40 of the reverse transcription solution engaged per reaction in a final volume of 10 μL. All reactions were carried out according to the manufacturer’s instructions. All primers are listed in Supplementary Table S1.

### 4.4. Western Blot

At day 3 post-transfection, cells were harvested for protein extraction. Cells were lysed and proteins recovered using RIPA buffer (150 mM NaCl, 1% TritonX-100, 0.5% sodium deoxycholate, 0.1% SDS and 50 mM Tris pH8) supplemented with 1 cOmplete Mini protease inhibitor cocktail (Roche) and 1 PhosSTOP phosphatase inhibitor cocktail (Roche). Next, 30 μg of proteins were loaded onto an 8% SDS-PAGE gel and subsequently transferred for 2 h at 240 mA onto a PVDF membrane (Millipore, Burlington, MA, USA). The membrane was blocked with 5% milk-TBS-Tween 0.1% for 1 h at RT, and then incubated overnight with anti-AGO1 antibody (D84G10, Cell Signaling Technology, Danvers, MA), which was diluted 1:1000 in 5% BSA-TBS-Tween 0.1%. The membrane was then washed 3X in TBS-Tween 0.1% and incubated at RT 1 h with a goat anti-rabbit antibody conjugated to HRP (ADI-SAB-300J, Enzo Life Sciences, Farmingdale, NY, USA), which was diluted 1:10,000 in BSA-TBS-Tween 0.1%. The membrane was washed 3X in TBS-Tween 0.1% and revealed using the SuperSignal West Pico PLUS Chemiluminescent substrate (Thermo Fisher) and CL-Xposure films (Life Technologies). For the detection of Vinculin (VCL), the membrane was stripped with 4X NaOH 0.4 M solution, blocked for 1 h at RT with 5% milk- TBS-Tween 0.1%, and incubated for 1 h with anti-VCL antibody diluted 1:100,000 (05386, Millipore).

### 4.5. GTEx Datasets

The median raw read counts of junctions of 52 normal tissues were obtained from the GTEx portal [49]. To define the expression of *AGO1-V2* and *AGO1*, we analyzed the junction read counts between their two first exons, which are specific to each of the two transcripts.

### 4.6. Processing of Public RNA-Seq Raw Data

Fastq files of the normal tissues (lung, testis, esophagus, colon, brain, and stomach), and all available cell lines of ESCA (n = 26), STAD (n = 37), and LUSC (n = 25) cancer types from the Cancer Cell Line Encyclopedia (CCLE) were downloaded from Sequence Read Archive (SRA) of NCBI [50,51]. All accession numbers are listed in Supplementary Table S2. FastQC v0.11.8 was used for read quality control [52]. The resulting files were mapped to the genome GRCh38 using HISAT2 v2.1.0 with default parameters [53] and converted to BAM files and indexed using Samtools v1.6 [54]. Read alignments corresponding to splice junctions were visualized using the Integrative Genomics Viewer v2.3.68 (IGV) [55]. The FeatureCounts tool was used for gene and exon quantification [56], which were expressed in Reads Per Kilobase per Million (RPKM). To determine the expression status of *AGO1* and *AGO1-V2*, we analyzed exon-level quantification of the first exon of the *AGO1* variant (ENSE00001459761) or the first exon of the *AGO1-V2* variant (ENSE00001459768). Cell lines were considered positive for *AGO1-V2* expression if the first exon of this variant displayed ≥0.5 RPKM.

### 4.7. NOA Testis RNA-Seq of Balagannavar’s Study

Fastq files of the control testis samples (normal or varicocele, n = 4) and the NOA testis samples (n = 8) were obtained from SRA [26] and processed as described above. All accession numbers are listed in Supplementary Table S2. NOA testis samples (n = 3) that nevertheless expressed germ cell markers *MAGEA1* and *MAGEA4* (≥0.5 RPKM) were discarded.

### 4.8. RNA-Seq of Cell Lines after DNA Demethylation Treatment

FastQ files of the IMR5-75 neuroblastoma cell line and the NCI-H146 small cell lung cancer cell line treated with 5-aza-2′-deoxycytidine 1 μM for 72 h (IMR5-75) or 0.5 μM for 96 h (NCI-H146) and controls treated with the solvent DMSO were downloaded from SRA [57,58] and processed as described above. Accession numbers are listed in Supplementary Table S2.

### 4.9. The Cancer Genome Atlas (TCGA) Consortium Datasets and Analyses

(1) Data collection: Normalized hg19 RNA-seq data with exon-level quantification and Infinium Human Methylation 450 K datasets for 21 cancer types for which we had expression data for at least 3 adjacent tissues were downloaded from The Cancer Genome Atlas (TCGA) consortium [59], using TCGAbiolinks v2.14.1 R-package [60]: BLCA (Bladder Urothelial Carcinoma), BRCA (Breast invasive carcinoma), CESC (Cervical squamous cell carcinoma and endocervical adenocarcinoma), CHOL (Cholangiocarcinoma), COAD (Colon adenocarcinoma), ESCA (Esophageal carcinoma), GBM (Glioblastoma multiforme), HNSC (Head and Neck squamous cell carcinoma), KICH (Kidney Chromophobe), KIRC (Kidney renal clear cell carcinoma), KIRP (Kidney renal papillary cell carcinoma), LIHC (Liver hepatocellular carcinoma), LUAD (Lung adenocarcinoma), LUSC (Lung squamous cell carcinoma), PAAD (Pancreatic adenocarcinoma), PCPG (Pheochromocytoma and Paraganglioma), PRAD (Prostate adenocarcinoma), READ (Rectum adenocarcinoma), STAD (Stomach adenocarcinoma), THCA (Thyroid carcinoma), and UCEC (Uterine Corpus Endometrial Carcinoma). Only unique primary (-01A) and metastatic (-06A) tumor samples, as well as unique normal adjacent tissues (-11A), were analyzed. (2) Expression analyses: To determine *AGO1* and *AGO1-V2* expression status, we analyzed RNA-seq exon-level quantification expressed as RPKM of the specific exon 1 of the AGO1 variant (ENSE00001459761) or the specific exon 1 of the *AGO1-V2* variant (ENSE00001459768). Samples were considered positive for *AGO1-V2* expression if the first exon of this variant displayed ≥ 0.5 RPKM. (3) DNA methylation analyses: For *AGO1* promoter DNA hypermethylation analyses, Infinium methylation levels (ß values) were examined for the 12 probes located in the promoter regions (TSS ± 1000 pb). We then selected the 8 CpG probes (cg13675280, cg06561166, cg01799338, cg27026509, cg11582116, cg01324793, cg03604172, and cg02141814) on the basis of data availability in all samples, and low mean methylation (≤40%) in normal adjacent tissues. For *AGO1-V2* promoter DNA hypomethylation analyses, Infinium methylation levels (ß values) were examined for the only CpG probe (cg09752398) located in the promoter region (TSS ± 1000 pb). We only analyzed samples where both exon-level quantification and Infinium methylation data were available.

### 4.10. DNA Methylation Datasets of Normal Tissues and Cell Lines

(1) Normal tissues: Bisulfite-seq data from normal human lung, testicular spermatozoa, esophagus, colon, brain, and stomach were visualized with the NIH Roadmap epigenomics track viewer [61]. (2) Cell lines: CpG clusters of Reduced Representation Bisulfite Sequencing (RRBS) DNA methylation data for all available cell lines of ESCA (n = 26), STAD (n = 37), and LUSC (n = 25) cancer types were obtained from the CCLE portal [62]. Files of CCLE RRBS CpG clusters (version 20181022), which include the average methylation levels for 47 CpGs in the *AGO1* promoter and 6 CpGs in the *AGO1-V2* promoter, were downloaded directly from the CCLE portal (https://sites.broadinstitute.org/ccle/datasets accessed on 22 March 2023).

### 4.11. Statistical Analysis and Graphical Representations

Statistical analysis was computed in R 4.3.0 (http://www.R-project.org). Graphs were generated using R packages ggplot2 (v3.4.3). The normality of the data was initially assessed using Quantile-Quantile (Q-Q) plots. The homogeneity of variances was tested with Fisher’s test. Depending on these preliminary assessments, either Student’s *t*-test or Welch’s t-test was performed. For paired samples, a paired *t*-test was used. Benjamini–Hochberg correction was used for the adjustment of *p*-values. All statistical analyses were conducted in R using the ‘stats’ package.

## Data Availability

Accessions to archived datasets analyzed in this study are listed in Table S2.

## Supplementary Materials

The following supporting information can be downloaded at: https://www.mdpi.com/article/10.3390/epigenomes8030024/s1, Figure S1: *AGO1* and *AGO1-V2* references and predicted structures, Figure S2: RT-qPCR analysis of *AGO1* and *AGO1V2* expression in various tumor cell lines. Figure S3: Distribution of CpG sites in the 5′ regions of *AGO1* and *AGO1-V2*, Table S1: siRNA sequences and qPCR primers and conditions, Table S2: Details and accessions of datasets used in the study.
