# Supplementary material for: DNA Hypomethylation Underlies Epigenetic Swapping between AGO1 and AGO1-V2 Isoforms in Tumors
Source: Epigenomes. 2024 Jun 22;8(3):24. doi: 10.3390/epigenomes8030024 (PMC11270204; doi:10.3390/epigenomes8030024)
Supplement: Supplementary file 1 [file epigenomes-08-00024-s001.zip › epigenomes-3025261-supplementary.pdf]

AGO1
mRNA: ENST00000373204.6
Protein isoform 1: Q9UL18

AGO1-V2
mRNA: ENST00000373206.5
Predicted protein isoform 2: B3KME0

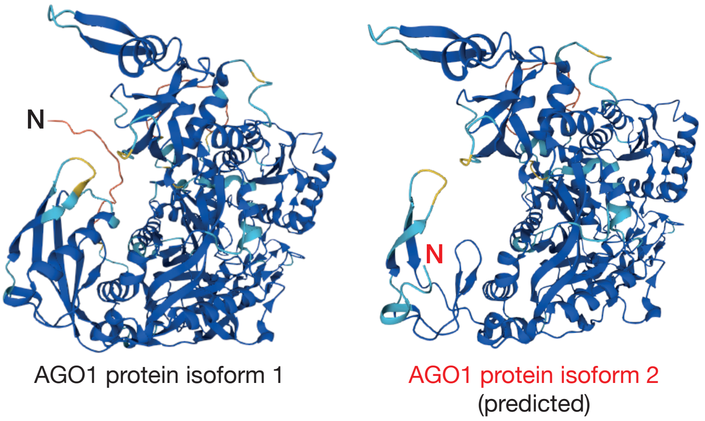

Figure S1. References of *AGO1* and *AGO1-V2* transcript variants (Ensembl) and corresponding proteins (UniProtKB). UniProtKB evidence for the existence AGO1 protein isoforms is: “at protein level“ for isoform 1, and “at transcript level“ for isoform 2. Predicted structures of the two AGO1 protein isoforms as reported by UniProtKB are presented, with color codes indicating model confidence (dark blue: very high; light blue: confident; yellow: low; orange: very low).

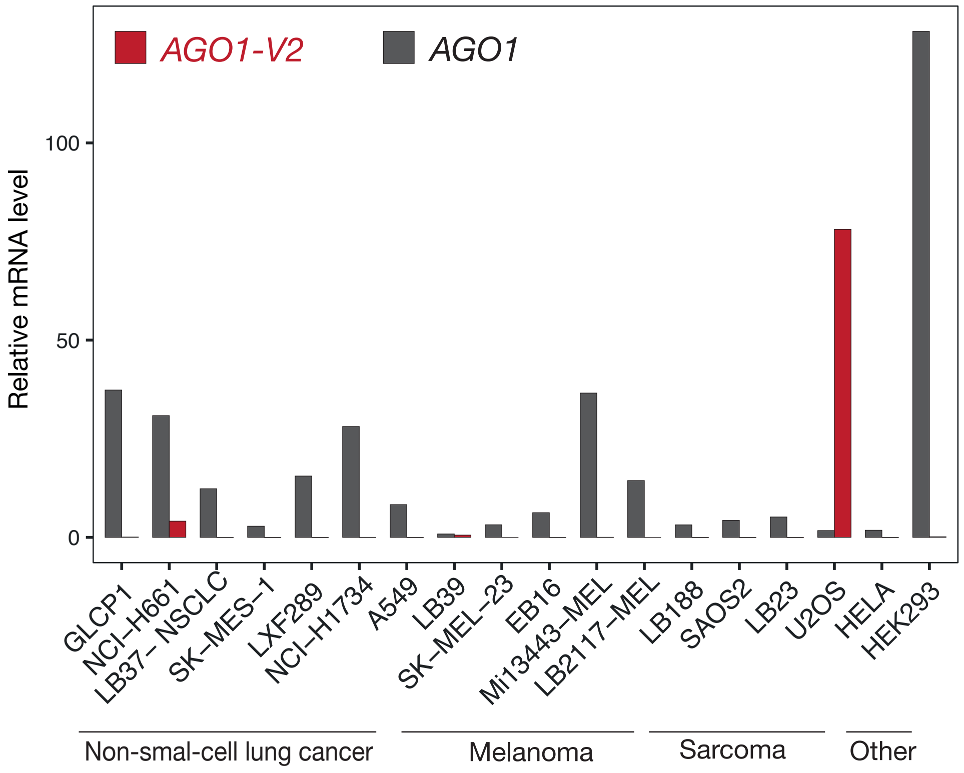

Figure S2. cDNA from various cell lines available in our laboratory were used in RT-qPCR experiments to evaluate expression of *AGO1-V2* and *AGO1* mRNAs (normalized to *ACTB* mRNA levels x 10,000). The cell lines were grouped according to their respective cancer types.

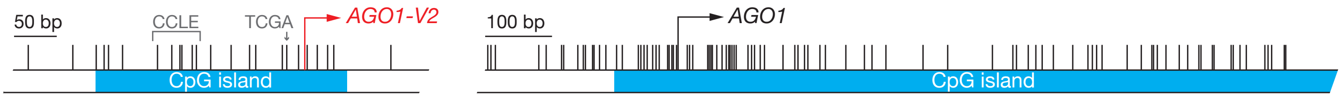

Figure S3. Distribution of CpG sites (vertical bars) around *AGO1-V2* and *AGO1* transcription start sites (broken arrows). Examination with the GC Profile 2.0 web tool identified DNA segments (296 bp for *AGO1-V2* and 1602 bp for *AGO1*) matching the criteria of a CpG island: G+C content >50%; observed/expected CpG ratio >0.60. For *AGO1-V2*, the CpG sites that are analyzed by RRBS in tumor cell lines (CCLE), or by Infinium methylation assay in tumor tissues (TCGA) are indicated.

Table S1. siRNA sequences and qPCR primers and conditions

siRNAs	Sequence (5'-3')
siLuc (Eurogentec)	CUUACGCUGAGUACUUCGA
siAGO1-all (Dharmacon)	pool 4 siRNAs - L004638-00-0020
siAGO1-V2 (Eurogentec)	AGGAGUGCGUGAGGCCCACGCAGAA

qPCR	Forward primer (5'-3')	Reverse primer (5'-3')	Additional information
ACTB	CCCTGGACTTCGAGCAAGAGAT	AAGGTAGTTTCGTGGATGCCACA	KAPA SYBR FAST (Annealing /Elongation: 30 sec at 60°C)
AGO1	CCTCACGCTGGACTTCACAGTCT	CTTGCGATCACCAAAGATCTGA	KAPA SYBR FAST (Annealing /Elongation: 30 sec at 60°C)
AGO1-V2	GCAAGCGCCTCAGGAGTGCGT	CTTGCGATCACCAAAGATCTGA	KAPA SYBR FAST (Annealing /Elongation: 30 sec at 60°C)

Fain et al. Supplementary materials

Table S2. Details and accession numbers of datasets used in the study

BioSample	Name	Accession number	BioProject	Layout	Instrument	File	Citation	Treatment
Normal tissue	Lung	SRR577579	PRJNA34535	PAIRED	Illumina HiSeq 2000	Fastq	Schultz MD et al., Nature, 2015	-
Normal tissue	Testis	ERR315415	PRJEB4337	PAIRED	Illumina HiSeq 2000	Fastq	Fagerberg L et al., Mol Cell Proteomics, 2014	-
Normal tissue	Esophagus	ERR315411	PRJEB4337	PAIRED	Illumina HiSeq 2000	Fastq	Fagerberg L et al., Mol Cell Proteomics, 2014	-
Normal tissue	Colon	ERR315357	PRJEB4337	PAIRED	Illumina HiSeq 2000	Fastq	Fagerberg L et al., Mol Cell Proteomics, 2014	-
Normal tissue	Brain	ERR315455	PRJEB4337	PAIRED	Illumina HiSeq 2000	Fastq	Fagerberg L et al., Mol Cell Proteomics, 2014	-
Normal tissue	Stomach	ERR315379	PRJEB4337	PAIRED	Illumina HiSeq 2000	Fastq	Fagerberg L et al., Mol Cell Proteomics, 2014	-
Commercial RNA from Asian, Normal	AN1	SRR9849353	PRJNA556935	PAIRED	Illumina HiSeq 2000	Fastq	Balagannavar F et al., Syst Biol Reprod Med, 2023	-
Commercial testicular RNA Caucasian Normal	CN1	SRR9849354	PRJNA556935	PAIRED	Illumina HiSeq 2000	Fastq	Balagannavar F et al., Syst Biol Reprod Med, 2023	-
RNA from biopsied testes, Indian donor 2, Varicocele	VA2	SRR9849355	PRJNA556935	PAIRED	Illumina HiSeq 2000	Fastq	Balagannavar F et al., Syst Biol Reprod Med, 2023	-
RNA from biopsied testes, Indian donor 1, Varicocele	VA1	SRR9849356	PRJNA556935	PAIRED	Illumina HiSeq 2000	Fastq	Balagannavar F et al., Syst Biol Reprod Med, 2023	-
RNA from biopsied testes, Indian donor 2, Non-Obstructive Azoospermia	NOA2	SRR9849357	PRJNA556935	PAIRED	Illumina HiSeq 2000	Fastq	Balagannavar F et al., Syst Biol Reprod Med, 2023	-
RNA from biopsied testes, Indian donor 1, Non-Obstructive Azoospermia	NOA1	SRR9849358	PRJNA556935	PAIRED	Illumina HiSeq 2000	Fastq	Balagannavar F et al., Syst Biol Reprod Med, 2023	-
RNA from biopsied testes, Indian donor 4, Non-Obstructive Azoospermia	NOA4	SRR9849359	PRJNA556935	PAIRED	Illumina HiSeq 2000	Fastq	Balagannavar F et al., Syst Biol Reprod Med, 2023	-
RNA from biopsied testes, Indian donor 3, Non-Obstructive Azoospermia	NOA3	SRR9849360	PRJNA556935	PAIRED	Illumina HiSeq 2000	Fastq	Balagannavar F et al., Syst Biol Reprod Med, 2023	-
RNA from biopsied testes, Indian donor 6, Non-Obstructive Azoospermia	NOA6	SRR9849361	PRJNA556935	PAIRED	Illumina HiSeq 2000	Fastq	Balagannavar F et al., Syst Biol Reprod Med, 2023	-
RNA from biopsied testes, Indian donor 5, Non-Obstructive Azoospermia	NOA5	SRR9849362	PRJNA556935	PAIRED	Illumina HiSeq 2000	Fastq	Balagannavar F et al., Syst Biol Reprod Med, 2023	-
RNA from biopsied testes, Indian donor 8, Non-Obstructive Azoospermia	NOA8	SRR9849363	PRJNA556935	PAIRED	Illumina HiSeq 2000	Fastq	Balagannavar F et al., Syst Biol Reprod Med, 2023	-
RNA from biopsied testes, Indian donor 7, Non-Obstructive Azoospermia	NOA7	SRR9849364	PRJNA556935	PAIRED	Illumina HiSeq 2000	Fastq	Balagannavar F et al., Syst Biol Reprod Med, 2023	-
Neuroblastoma cells lines	IMR5-75	SRR3326020	PRJNA317239	PAIRED	Illumina HiSeq 2000	Fastq	Henrich KO et al., Cancer Res, 2016	DMSO, 72 hours
Neuroblastoma cells lines	IMR5-75	SRR3326021	PRJNA317239	PAIRED	Illumina HiSeq 2000	Fastq	Henrich KO et al., Cancer Res, 2016	DMSO, 72 hours
Neuroblastoma cells lines	IMR5-75	SRR3326022	PRJNA317239	PAIRED	Illumina HiSeq 2000	Fastq	Henrich KO et al., Cancer Res, 2016	Aza; 1 μM, 72 hours
Neuroblastoma cells lines	IMR5-75	SRR3326023	PRJNA317239	PAIRED	Illumina HiSeq 2000	Fastq	Henrich KO et al., Cancer Res, 2016	Aza; 1 μM, 72 hours
Small cell lung carcinoma cells lines	NCI-H146	SRR25387785	PRJNA997364	PAIRED	Illumina HiSeq 1500	Fastq	Biswas S et al., Cell Rep, 2023	DMSO, 96 hours
Small cell lung carcinoma cells lines	NCI-H146	SRR25387783	PRJNA997364	PAIRED	Illumina HiSeq 1500	Fastq	Biswas S et al., Cell Rep, 2023	DMSO, 96 hours
Small cell lung carcinoma cells lines	NCI-H146	SRR25387782	PRJNA997364	PAIRED	Illumina HiSeq 1500	Fastq	Biswas S et al., Cell Rep, 2023	Aza; 0.5 μM, 96 hours
Small cell lung carcinoma cells lines	NCI-H146	SRR25387784	PRJNA997364	PAIRED	Illumina HiSeq 1500	Fastq	Biswas S et al., Cell Rep, 2023	Aza; 0.5 μM, 96 hours
LUSC cell lines (CCLE)	Calu-1	SRR8615533	PRJNA523380	PAIRED	Illumina HiSeq 2500	Fastq	Ghandi M et al., Nature, 2019	-
LUSC cell lines (CCLE)	EBC-1	SRR8615634	PRJNA523380	PAIRED	Illumina HiSeq 2500	Fastq	Ghandi M et al., Nature, 2019	-
LUSC cell lines (CCLE)	EPLC-272H	SRR8616217	PRJNA523380	PAIRED	Illumina HiSeq 2500	Fastq	Ghandi M et al., Nature, 2019	-
LUSC cell lines (CCLE)	HARA	SRR8615820	PRJNA523380	PAIRED	Illumina HiSeq 2500	Fastq	Ghandi M et al., Nature, 2019	-
LUSC cell lines (CCLE)	HCC-15	SRR8616176	PRJNA523380	PAIRED	Illumina HiSeq 2500	Fastq	Ghandi M et al., Nature, 2019	-
LUSC cell lines (CCLE)	HCC-1588	SRR8616182	PRJNA523380	PAIRED	Illumina HiSeq 2500	Fastq	Ghandi M et al., Nature, 2019	-
LUSC cell lines (CCLE)	HCC-2814	SRR8615997	PRJNA523380	PAIRED	Illumina HiSeq 2500	Fastq	Ghandi M et al., Nature, 2019	-
LUSC cell lines (CCLE)	HCC-95	SRR8615283	PRJNA523380	PAIRED	Illumina HiSeq 2500	Fastq	Ghandi M et al., Nature, 2019	-
LUSC cell lines (CCLE)	HLF-a	SRR8616136	PRJNA523380	PAIRED	Illumina HiSeq 2500	Fastq	Ghandi M et al., Nature, 2019	-
LUSC cell lines (CCLE)	KNS-62	SRR8615709	PRJNA523380	PAIRED	Illumina HiSeq 2500	Fastq	Ghandi M et al., Nature, 2019	-
LUSC cell lines (CCLE)	LC-1F	SRR8616103	PRJNA523380	PAIRED	Illumina HiSeq 2500	Fastq	Ghandi M et al., Nature, 2019	-
LUSC cell lines (CCLE)	LK-2	SRR8615840	PRJNA523380	PAIRED	Illumina HiSeq 2500	Fastq	Ghandi M et al., Nature, 2019	-
LUSC cell lines (CCLE)	LOU-NH91	SRR8615550	PRJNA523380	PAIRED	Illumina HiSeq 2500	Fastq	Ghandi M et al., Nature, 2019	-
LUSC cell lines (CCLE)	LUDLU-1	SRR8615402	PRJNA523380	PAIRED	Illumina HiSeq 2500	Fastq	Ghandi M et al., Nature, 2019	-
LUSC cell lines (CCLE)	NCI-H1385	SRR8615616	PRJNA523380	PAIRED	Illumina HiSeq 2500	Fastq	Ghandi M et al., Nature, 2019	-
LUSC cell lines (CCLE)	NCI-H1869	SRR8615615	PRJNA523380	PAIRED	Illumina HiSeq 2500	Fastq	Ghandi M et al., Nature, 2019	-
LUSC cell lines (CCLE)	NCI-H2170	SRR8616108	PRJNA523380	PAIRED	Illumina HiSeq 2500	Fastq	Ghandi M et al., Nature, 2019	-
LUSC cell lines (CCLE)	NCI-H226	SRR8616114	PRJNA523380	PAIRED	Illumina HiSeq 2500	Fastq	Ghandi M et al., Nature, 2019	-
LUSC cell lines (CCLE)	NCI-H520	SRR8615799	PRJNA523380	PAIRED	Illumina HiSeq 2500	Fastq	Ghandi M et al., Nature, 2019	-
LUSC cell lines (CCLE)	RERF-LC-AI	SRR8615591	PRJNA523380	PAIRED	Illumina HiSeq 2500	Fastq	Ghandi M et al., Nature, 2019	-
LUSC cell lines (CCLE)	RERF-LC-Sq1	SRR8615797	PRJNA523380	PAIRED	Illumina HiSeq 2500	Fastq	Ghandi M et al., Nature, 2019	-
LUSC cell lines (CCLE)	SK-MES-1	SRR8616057	PRJNA523380	PAIRED	Illumina HiSeq 2500	Fastq	Ghandi M et al., Nature, 2019	-
LUSC cell lines (CCLE)	Sq-1	SRR8616184	PRJNA523380	PAIRED	Illumina HiSeq 2500	Fastq	Ghandi M et al., Nature, 2019	-
LUSC cell lines (CCLE)	SW 1573	SRR8615561	PRJNA523380	PAIRED	Illumina HiSeq 2500	Fastq	Ghandi M et al., Nature, 2019	-
LUSC cell lines (CCLE)	SW 900	SRR8615853	PRJNA523380	PAIRED	Illumina HiSeq 2500	Fastq	Ghandi M et al., Nature, 2019	-
ESCA cell lines (CCLE)	EC-GI-10	SRR8615274	PRJNA523380	PAIRED	Illumina HiSeq 2500	Fastq	Ghandi M et al., Nature, 2019	-
ESCA cell lines (CCLE)	OE19	SRR8615226	PRJNA523380	PAIRED	Illumina HiSeq 2500	Fastq	Ghandi M et al., Nature, 2019	-
ESCA cell lines (CCLE)	JH-EsoAd1	SRR8615484	PRJNA523380	PAIRED	Illumina HiSeq 2500	Fastq	Ghandi M et al., Nature, 2019	-
ESCA cell lines (CCLE)	COLO-680N	SRR8615991	PRJNA523380	PAIRED	Illumina HiSeq 2500	Fastq	Ghandi M et al., Nature, 2019	-
ESCA cell lines (CCLE)	KYSE-140	SRR8616035	PRJNA523380	PAIRED	Illumina HiSeq 2500	Fastq	Ghandi M et al., Nature, 2019	-
ESCA cell lines (CCLE)	KYSE-150	SRR8616034	PRJNA523380	PAIRED	Illumina HiSeq 2500	Fastq	Ghandi M et al., Nature, 2019	-
ESCA cell lines (CCLE)	KYSE-180	SRR8616037	PRJNA523380	PAIRED	Illumina HiSeq 2500	Fastq	Ghandi M et al., Nature, 2019	-
ESCA cell lines (CCLE)	KYSE-270	SRR8616036	PRJNA523380	PAIRED	Illumina HiSeq 2500	Fastq	Ghandi M et al., Nature, 2019	-
ESCA cell lines (CCLE)	KYSE-30	SRR8616039	PRJNA523380	PAIRED	Illumina HiSeq 2500	Fastq	Ghandi M et al., Nature, 2019	-
ESCA cell lines (CCLE)	KYSE-410	SRR8616038	PRJNA523380	PAIRED	Illumina HiSeq 2500	Fastq	Ghandi M et al., Nature, 2019	-
ESCA cell lines (CCLE)	KYSE-450	SRR8615511	PRJNA523380	PAIRED	Illumina HiSeq 2500	Fastq	Ghandi M et al., Nature, 2019	-
ESCA cell lines (CCLE)	KYSE-510	SRR8615500	PRJNA523380	PAIRED	Illumina HiSeq 2500	Fastq	Ghandi M et al., Nature, 2019	-
ESCA cell lines (CCLE)	KYSE-520	SRR8615481	PRJNA523380	PAIRED	Illumina HiSeq 2500	Fastq	Ghandi M et al., Nature, 2019	-
ESCA cell lines (CCLE)	KYSE-70	SRR8615482	PRJNA523380	PAIRED	Illumina HiSeq 2500	Fastq	Ghandi M et al., Nature, 2019	-
ESCA cell lines (CCLE)	OE21	SRR8615227	PRJNA523380	PAIRED	Illumina HiSeq 2500	Fastq	Ghandi M et al., Nature, 2019	-
ESCA cell lines (CCLE)	TE-1	SRR8615464	PRJNA523380	PAIRED	Illumina HiSeq 2500	Fastq	Ghandi M et al., Nature, 2019	-
ESCA cell lines (CCLE)	TE-10	SRR8615827	PRJNA523380	PAIRED	Illumina HiSeq 2500	Fastq	Ghandi M et al., Nature, 2019	-
ESCA cell lines (CCLE)	TE-11	SRR8615828	PRJNA523380	PAIRED	Illumina HiSeq 2500	Fastq	Ghandi M et al., Nature, 2019	-
ESCA cell lines (CCLE)	TE-14	SRR8615834	PRJNA523380	PAIRED	Illumina HiSeq 2500	Fastq	Ghandi M et al., Nature, 2019	-
ESCA cell lines (CCLE)	TE-15	SRR8615222	PRJNA523380	PAIRED	Illumina HiSeq 2500	Fastq	Ghandi M et al., Nature, 2019	-
ESCA cell lines (CCLE)	TE-4	SRR8615470	PRJNA523380	PAIRED	Illumina HiSeq 2500	Fastq	Ghandi M et al., Nature, 2019	-
ESCA cell lines (CCLE)	TE-5	SRR8615469	PRJNA523380	PAIRED	Illumina HiSeq 2500	Fastq	Ghandi M et al., Nature, 2019	-
ESCA cell lines (CCLE)	TE-6	SRR8615467	PRJNA523380	PAIRED	Illumina HiSeq 2500	Fastq	Ghandi M et al., Nature, 2019	-
ESCA cell lines (CCLE)	TE-8	SRR8615390	PRJNA523380	PAIRED	Illumina HiSeq 2500	Fastq	Ghandi M et al., Nature, 2019	-
ESCA cell lines (CCLE)	TE-9	SRR8616128	PRJNA523380	PAIRED	Illumina HiSeq 2500	Fastq	Ghandi M et al., Nature, 2019	-
ESCA cell lines (CCLE)	OE33	SRR8615228	PRJNA523380	PAIRED	Illumina HiSeq 2500	Fastq	Ghandi M et al., Nature, 2019	-
STAD cell lines (CCLE)	Hs 746T	SRR8615322	PRJNA523380	PAIRED	Illumina HiSeq 2500	Fastq	Ghandi M et al., Nature, 2019	-
STAD cell lines (CCLE)	HuG1-N	SRR8615665	PRJNA523380	PAIRED	Illumina HiSeq 2500	Fastq	Ghandi M et al., Nature, 2019	-
STAD cell lines (CCLE)	KE-39	SRR8615366	PRJNA523380	PAIRED	Illumina HiSeq 2500	Fastq	Ghandi M et al., Nature, 2019	-
STAD cell lines (CCLE)	NCC-StC-K140	SRR8615342	PRJNA523380	PAIRED	Illumina HiSeq 2500	Fastq	Ghandi M et al., Nature, 2019	-
STAD cell lines (CCLE)	NCI-N87	SRR8615495	PRJNA523380	PAIRED	Illumina HiSeq 2500	Fastq	Ghandi M et al., Nature, 2019	-
STAD cell lines (CCLE)	NUGC-3	SRR8615450	PRJNA523380	PAIRED	Illumina HiSeq 2500	Fastq	Ghandi M et al., Nature, 2019	-
STAD cell lines (CCLE)	RERF-GC-1B	SRR8615596	PRJNA523380	PAIRED	Illumina HiSeq 2500	Fastq	Ghandi M et al., Nature, 2019	-
STAD cell lines (CCLE)	SH-10-TC	SRR8615744	PRJNA523380	PAIRED	Illumina HiSeq 2500	Fastq	Ghandi M et al., Nature, 2019	-
STAD cell lines (CCLE)	SNU-216	SRR8615309	PRJNA523380	PAIRED	Illumina HiSeq 2500	Fastq	Ghandi M et al., Nature, 2019	-
STAD cell lines (CCLE)	SNU-520	SRR8615658	PRJNA523380	PAIRED	Illumina HiSeq 2500	Fastq	Ghandi M et al., Nature, 2019	-
STAD cell lines (CCLE)	SNU-601	SRR8616025	PRJNA523380	PAIRED	Illumina HiSeq 2500	Fastq	Ghandi M et al., Nature, 2019	-
STAD cell lines (CCLE)	SNU-620	SRR8616027	PRJNA523380	PAIRED	Illumina HiSeq 2500	Fastq	Ghandi M et al., Nature, 2019	-
STAD cell lines (CCLE)	SNU-719	SRR8616031	PRJNA523380	PAIRED	Illumina HiSeq 2500	Fastq	Ghandi M et al., Nature, 2019	-
STAD cell lines (CCLE)	TGBC11TKB	SRR8615672	PRJNA523380	PAIRED	Illumina HiSeq 2500	Fastq	Ghandi M et al., Nature, 2019	-
STAD cell lines (CCLE)	23132/87	SRR8616168	PRJNA523380	PAIRED	Illumina HiSeq 2500	Fastq	Ghandi M et al., Nature, 2019	-
STAD cell lines (CCLE)	AGS	SRR8615404	PRJNA523380	PAIRED	Illumina HiSeq 2500	Fastq	Ghandi M et al., Nature, 2019	-
STAD cell lines (CCLE)	GCIY	SRR8615733	PRJNA523380	PAIRED	Illumina HiSeq 2500	Fastq	Ghandi M et al., Nature, 2019	-
STAD cell lines (CCLE)	GSS	SRR8615379	PRJNA523380	PAIRED	Illumina HiSeq 2500	Fastq	Ghandi M et al., Nature, 2019	-
STAD cell lines (CCLE)	GSU	SRR8615389	PRJNA523380	PAIRED	Illumina HiSeq 2500	Fastq	Ghandi M et al., Nature, 2019	-
STAD cell lines (CCLE)	KATO III	SRR8615362	PRJNA523380	PAIRED	Illumina HiSeq 2500	Fastq	Ghandi M et al., Nature, 2019	-
STAD cell lines (CCLE)	LMSU	SRR8615839	PRJNA523380	PAIRED	Illumina HiSeq 2500	Fastq	Ghandi M et al., Nature, 2019	-
STAD cell lines (CCLE)	NUGC-2	SRR8615449	PRJNA523380	PAIRED	Illumina HiSeq 2500	Fastq	Ghandi M et al., Nature, 2019	-
STAD cell lines (CCLE)	FU97	SRR8615735	PRJNA523380	PAIRED	Illumina HiSeq 2500	Fastq	Ghandi M et al., Nature, 2019	-
STAD cell lines (CCLE)	MKN-45	SRR8615515	PRJNA523380	PAIRED	Illumina HiSeq 2500	Fastq	Ghandi M et al., Nature, 2019	-
STAD cell lines (CCLE)	OCUM-1	SRR8615225	PRJNA523380	PAIRED	Illumina HiSeq 2500	Fastq	Ghandi M et al., Nature, 2019	-
STAD cell lines (CCLE)	IM95	SRR8615841	PRJNA523380	PAIRED	Illumina HiSeq 2500	Fastq	Ghandi M et al., Nature, 2019	-
STAD cell lines (CCLE)	MKN1	SRR8615518	PRJNA523380	PAIRED	Illumina HiSeq 2500	Fastq	Ghandi M et al., Nature, 2019	-
STAD cell lines (CCLE)	NUGC-4	SRR8615244	PRJNA523380	PAIRED	Illumina HiSeq 2500	Fastq	Ghandi M et al., Nature, 2019	-
STAD cell lines (CCLE)	SNU-668	SRR8616029	PRJNA523380	PAIRED	Illumina HiSeq 2500	Fastq	Ghandi M et al., Nature, 2019	-
STAD cell lines (CCLE)	ECC10	SRR8615633	PRJNA523380	PAIRED	Illumina HiSeq 2500	Fastq	Ghandi M et al., Nature, 2019	-
STAD cell lines (CCLE)	ECC12	SRR8615624	PRJNA523380	PAIRED	Illumina HiSeq 2500	Fastq	Ghandi M et al., Nature, 2019	-
STAD cell lines (CCLE)	MKN7	SRR8616068	PRJNA523380	PAIRED	Illumina HiSeq 2500	Fastq	Ghandi M et al., Nature, 2019	-
STAD cell lines (CCLE)	MKN74	SRR8615516	PRJNA523380	PAIRED	Illumina HiSeq 2500	Fastq	Ghandi M et al., Nature, 2019	-
STAD cell lines (CCLE)	HGC-27	SRR8616131	PRJNA523380	PAIRED	Illumina HiSeq 2500	Fastq	Ghandi M et al., Nature, 2019	-
STAD cell lines (CCLE)	SNU-1	SRR8615933	PRJNA523380	PAIRED	Illumina HiSeq 2500	Fastq	Ghandi M et al., Nature, 2019	-
STAD cell lines (CCLE)	SNU-16	SRR8615934	PRJNA523380	PAIRED	Illumina HiSeq 2500	Fastq	Ghandi M et al., Nature, 2019	-
STAD cell lines (CCLE)	SNU-5	SRR8615659	PRJNA523380	PAIRED	Illumina HiSeq 2500	Fastq	Ghandi M et al., Nature, 2019	-
